# Dynamic contrast enhanced high field magnetic resonance imaging for canine primary intracranial neoplasia

**DOI:** 10.3389/fvets.2024.1468831

**Published:** 2024-10-04

**Authors:** Simon Choi, Caterina Brighi, Sam Long

**Affiliations:** ^1^Veterinary Referral Hospital, Dandenong, VIC, Australia; ^2^Image X Institute, Sydney School of Health Services, The University of Sydney, Sydney, NSW, Australia

**Keywords:** dynamic contrast-enhanced MRI, meningioma, Ktrans, cerebral blood flow, perfusion MRI

## Abstract

**Introduction:**

Distinguishing meningiomas from other intracranial neoplasms is clinically relevant as the prognostic and therapeutic implications differ greatly and influence clinical decision making. Dynamic contrast-enhanced MRI (DCE-MRI) is an imaging technique that assists with characterisation of physiologic alterations such as blood flow and tissue vascular permeability. Quantitative pharmacokinetic analysis utilising DCE-MRI has not been studied in canine neuro-oncology.

**Methods:**

A retrospective study was performed in canine patients that underwent DCE-MRI with an imaging diagnosis of an intracranial meningioma and surgery for histopathological diagnosis. Kinetic parameters Ktrans and cerebral blood flow were measured and compared to assess whether differences could be identified between meningiomas and other intracranial neoplasms.

**Results:**

Six dogs with meningiomas and 3 dogs with other intracranial neoplasms were included for statistical analysis. Cerebral blood flow values were found to be statistically higher within meningiomas compared to other intracranial neoplasms. Ktrans values were higher within meningiomas than in other types of intracranial tumours, however this difference did not reach statistical significance.

**Discussion:**

Based on the results of this study cerebral blood flow measurement can be utilised to differentiate canine intracranial meningiomas from other similar appearing intracranial tumours.

## Introduction

Brain tumours comprise one of the most common diseases diagnosed in older dogs, with an incidence of approximately 14–15 per 100,000 dogs at risk (1) and being diagnosed in 4.5% of all dogs undergoing *post mortem* examination (2, 3). Meningiomas are the most common primary intracranial neoplasm in dogs (4–7). The majority of these are benign tumours and cytoreductive surgery alone can result in median survival times of 7–12 months, or 16–30 months with the addition of radiation therapy (3, 8, 9) whilst with palliative treatment (corticosteroids and anticonvulsants) mean survival times ranging from 59 to 81 days have been reported (10, 11). For this reason, identifying these tumours is important for the clinician since they potentially represent one of the more treatable canine intracranial neoplasms.

Meningiomas are most frequently observed in the olfactory and frontal regions although can also be found in the cerebral or cerebellar convexity, optic chiasm, suprasellar and parasellar regions, cerebellopontine, basilar, tentorial, falcine, foraminal or intraventricular locations (6, 12). On Magnetic Resonance Imaging (MRI) meningiomas usually demonstrate uniform T1-weighted (T1W) isointensity but can occasionally be hypo-or hyperintense. Roughly 70% are T2-weighted (T2W) hyperintense with the remainder being isointense (4, 12). 60–70% demonstrate marked uniform contrast enhancement with the rest being more heterogenous. Following contrast enhancement well-defined tumour margins, an overall spherical, ovoid or globular shape along with a broad-based external margin which conforms to the meningeal plane aid in recognising these mass lesions as meningeal in origin. Dural tail sign (thickening of the meninges adjacent to the tumour) is recognised as a frequent feature although not pathognomonic for meningiomas. The adjacent calvarium may demonstrate reactive hyperostosis which is also a sign commonly associated with meningiomas (4).

The sensitivity of MRI to accurately identify intracranial meningiomas in dogs ranges between 66 and 100% (6, 13). However, other canine intracranial tumours can demonstrate MRI appearances that closely mimic meningiomas including intracranial histiocytic sarcoma (14, 15), lymphoma (16), intracranial germ cell tumours (15), granular cell tumours (17–19), olfactory neuroblastoma (20) and pituitary tumours (21). Many of these are biologically aggressive tumours compared to meningiomas and thus their response to treatment and prognosis differs greatly (18, 22, 23). Ultimately the acquisition of biopsy specimens for histopathological examination and immunohistochemical preparations remains the best way to achieve a definitive diagnosis. These tissue-based analyses however are associated with practical and financial constraints. Several frame-based and frameless stereotactic brain biopsy techniques have been described [for more recent examples see references (24–28)]. Although these newer techniques can achieve high precision and increase the likelihood of a diagnostic biopsy specimen there are several limitations. These procedures remain costly with frame-based procedures often requiring multiple anaesthetics to complete the workflow in addition to additional surgery that is required for placement of headframes or surgical head clamps for some frameless platforms (25, 28). Furthermore biopsy-associated clinical morbidity and mortality remain potential risks (24, 26). There remains, therefore, a need for noninvasive imaging techniques to improve the ability to predict tumour type and aid in subsequent appropriate choice of therapy.

Perfusion-weighted imaging is the study of blood flow in the capillary networks of tissues and the exchanges between blood and extravascular space (29). This allows the early detection and characterisation of a lesion and in recent years this approach has been used and developed further to optimise treatments and monitor the clinical course of lesions throughout treatment. Patterns of perfusion-weighted imaging have also been used to distinguish between different tumour types in MRI imaging studies (30). A widely used strategy to assess this perfusion is through the analysis of the kinetics of passage of a bolus of paramagnetic, low-molecular weight contrast agent through tissue after an intravenous injection (31). When this contrast medium is intravenously injected, it enters tumour blood vessels before leaking into extra-vascular extracellular space (in tumours, 12–45% of contrast medium is reported to leak into extracellular space during first pass) and subsequently diffusing within the interstitial space.

In clinical settings contrast-enhanced MRI is routinely performed through the intravenous administration of a paramagnetic gadolinium-based contrast medium. Conventionally however only post-contrast T1-weighted images are routinely acquired which provides a single timepoint of tumour enhancement. Dynamic contrast-enhanced MRI (DCE-MRI) involves the acquisition of fast, repeated images before, during and after the rapid administration of intravenous contrast medium. The resultant temporal change in signal intensity within the tumour is reflective of tumour perfusion, vessel permeability and the extravascular-extracellular environment, providing information not only of morphology but also enabling quantitative assessment of physiologic alterations (29).

DCE-MRI is also referred to as permeability MRI and provides quantitative assessment of tissue perfusion and blood–brain barrier dysfunction (23) allowing for non-invasive, quantitative assessment of tissue vessel density, integrity and permeability. This information can then be used to assess angiogenesis, hypoxia and interpreting various biomarkers (22). Previous studies in humans have used DCE MRI to distinguish between lower and higher-grade tumours along with differentiation of meningiomas from other dural-based neoplasms (30, 32).

Perfusion parameters such as Ktrans and cerebral blood flow (rBF) are commonly assessed in humans to quantify microvascularity of brain neoplasms (31). Ktrans is a term which denotes the rate at which contrast agent is delivered to the extravascular, extracellular space (EES) per volume of tissue and contrast agent concentration in the arterial blood plasma (33). It reflects transendothelial transport of contrast medium from the vascular compartment to the tumour insterstitium (i.e., vessel permeability). rBF is a perfusion parameter that is used to quantify the increased microvascularity of brain neoplasms (34).

A scant number of veterinary studies have utilised DCE-MRI for canine brain tumours. One paper assessed DCE-MRI with the intent of characterising perfusion parameters as a function of tumour type (35) and another aimed at assessing blood–brain barrier dysfunction in dogs with brain tumours using subtraction enhancement analysis (36).

The aim of this study was to evaluate the use of DCE-MRI in canine brain tumours and to perform quantitative pharmacokinetic modelling to differentiate between meningiomas and other intracranial tumours with similar MRI appearance.

## Materials and methods

### Study population

Animals presenting to a private neurology referral service that underwent conventional brain MRI in addition to DCE MRI between 2019 and 2021 were included. Patient inclusion criteria were: (1) Complete brain MRI study with standard and DCE MRI sequences performed, (2) Imaging diagnosis of an intracranial meningioma by a board-certified specialist in diagnostic imaging, and (3). Craniotomy/craniectomy for surgical resection and subsequent tissue histopathological assessment by a board-certified pathologist. The following medical record data was recorded: age, breed, sex, medical history, complete physical and neurological examination, complete blood count (CBC) and biochemical profile.

### Image acquisition and image review

Dogs were premedicated with butorphanol (0.2 mg/kg intravenously) and anaesthesia was induced with midazolam and alfaxolone co-induction. Maintenance of anaesthesia was provided by isoflurane and oxygen. Dogs were placed in dorsal recumbency for MR imaging of the brain.

Dogs were imaged using a 3.0 T Siemens Magnetom Skyra (Siemens Healthcare GmbH) MRI scanner. Images obtained included sagittal and transverse T2-weighted (T2W) sequences, fluid-attenuated inversion recovery (FLAIR), susceptibility weighted imaging (T2*), apparent diffusion coefficient and diffusion weighted sequences in transverse plane and T1-weighted (T1 MP-RAGE) sequences. A T1 mapping sequence was acquired before the DCE sequence to obtain native T1 maps. This sequence was used to acquire the DCE-MRI data in transverse planes. DCE-MRI involved TWIST pulse sequence acquired before, during and after the administration of gadolinium bolus using the following sequence of parameters: repetition time (TR) = 4.83 ms; echo time (TE) = 1.87 ms; flip angle: 12 degrees; number of excitation (NEX) = 2, phase field-of-view (pFOV) = 260 mm; base resolution 192, phase resolution 69%; phase direction: anterior to posterior (ventral to dorsal), acceleration factor: 2, field of view (FOV) = 260 mm; and slice thickness = 3 mm with 70 measurements. Voxel size: 1.4 mm × 1.4 mm × 3.0 mm. A dose of 0.1 mmol/kg gadopentetate dimeglumine (Gd-DTPA; *Magnevist – Bayer Australia Ltd*) was used as the paramagnetic contrast agent, given as a bolus after the first acquisition. For TWIST sequences 10 measurements were acquired at 5, 10, 15, 20, and 30 s following contrast administration.

**Figure 1:**
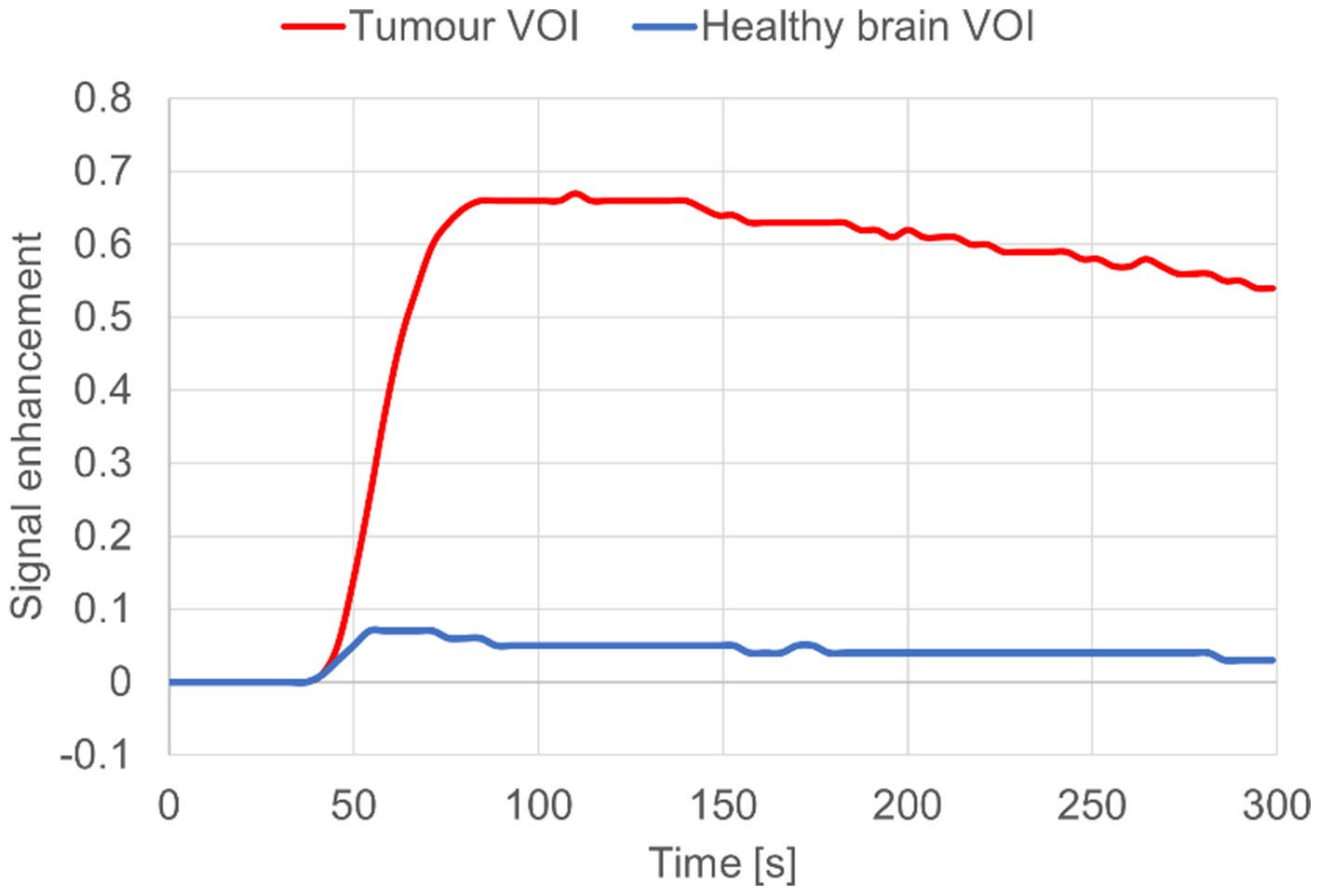
Signal enhancement curve for dog 8 diagnosed with meningioma. Tumour CE VOI (red) vs. Contralateral brain VOI “control” (blue).

**Figure 2:**
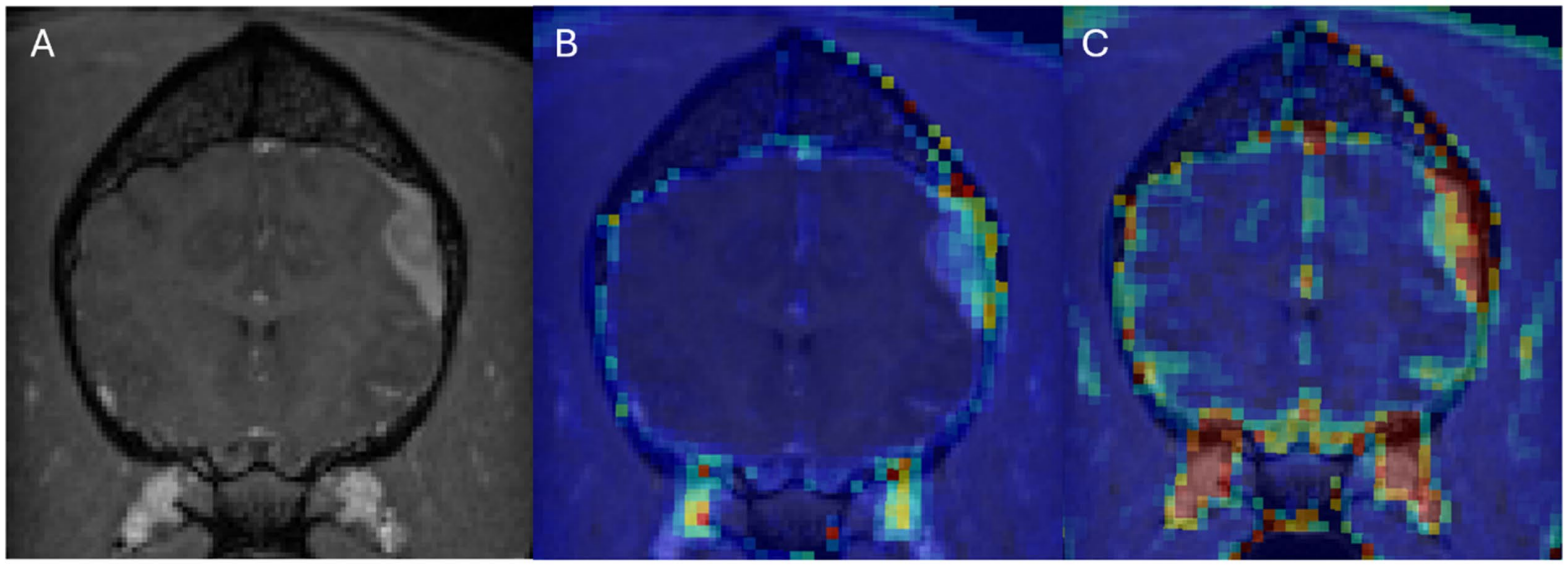
Dog 8 with meningioma. Transverse T1w post-contrast **(A)**, Transverse T1w post-contrast with Ktrans map overlay **(B)**, Transverse T1w post-contrast with rBF map overlay **(C)**.

**Figure 3:**
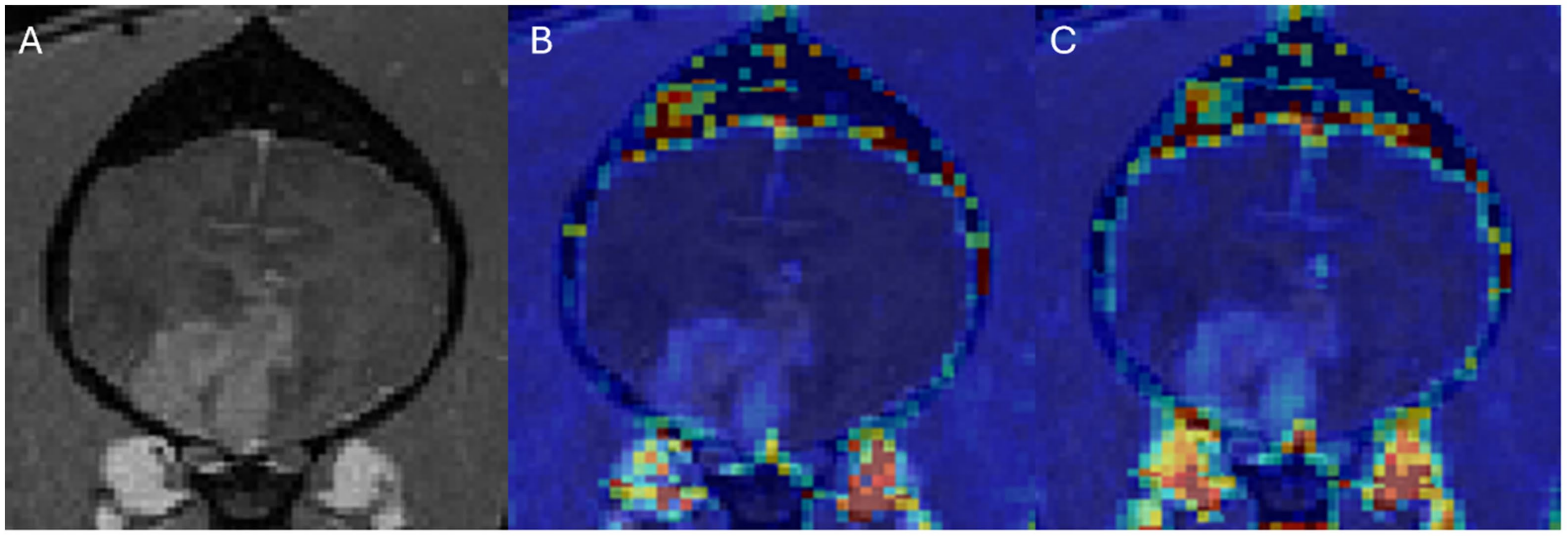
Dog 2 with histiocytic sarcoma. Transverse T1w post-contrast **(A)**, Transverse T1w post-contrast with Ktrans map overlay **(B)**, Transverse T1w post-contrast with rBF map overlay **(C)**.

**Figure 4:**
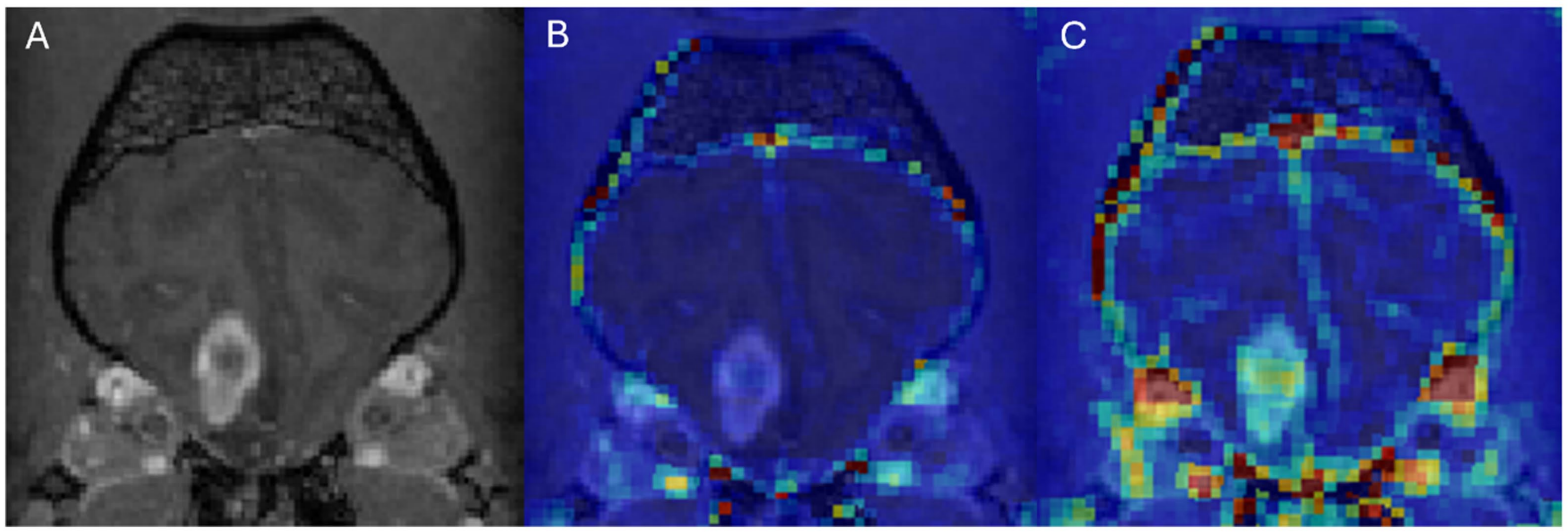
Dog 7 with glial cell neoplasm. Transverse T1w post-contrast **(A)**, Transverse T1w post-contrast with Ktrans map overlay **(B)**, Transverse T1w post-contrast with rBF map overlay **(C)**.

**Figure 5:**
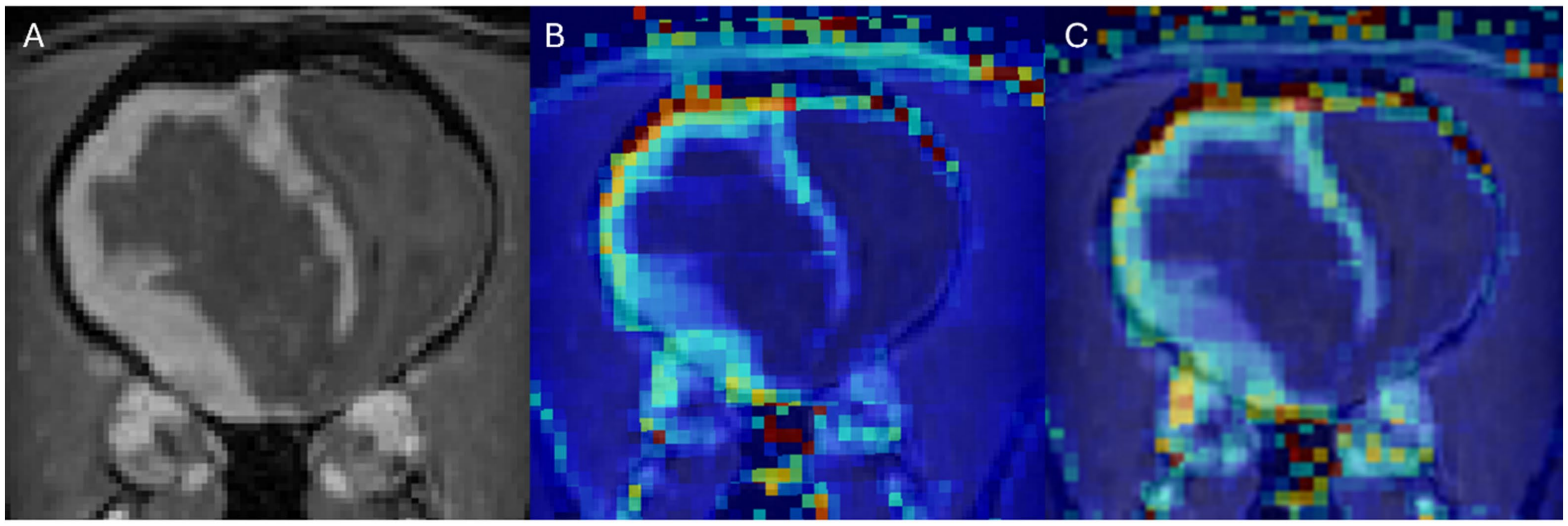
Dog 10 with granular cell tumour. Transverse T1w post-contrast **(A)**, Transverse T1w post-contrast with Ktrans map overlay **(B)**, Transverse T1w post-contrast with rBF map overlay **(C)**.

In DCE-MRI the signal acquired is used to generate a time intensity curve for a region of interest representing the tissue’s response to the arrival of contrast agent in enhancement values (Figure 1). Each post contrast sequence, representing different time points, is analysed for induced variation in signal intensity and parametric maps of permeability (Ktrans) and cerebral blood flow (rBF) are generated by voxel-wise modelling of this data (37). Cerebral blood flow (rBF) is determined using cerebral blood volume divided by mean transit time, where area under the signal versus time curve is used to approximate cerebral blood volume (26). rBF maps were calculated by fitting the pixel data to the two-compartment uptake model, with T1 and partial-volume correction (Figures 2–5).

DICOM images were converted into NIFTI format using dcm2niix software tool [dcm2niix, (38)]. T1 maps were derived from the T1 mapping sequence using the dedicated tool in the Nordic-ICE software package (NordicNeuroLab, Bergen, Norway) used for dynamic image analysis. T1 maps and DCE images were then linearly resampled to the T1-contrast enhanced (T1-CE) image using the software ITK-SNAP (39). Binary masks of the tumour volume of interest (VOI) and contrast enhancing (CE) tumour VOI were manually delineated on the T1-CE images, using a semi-automatic active contour segmentation tool (ITK-SNAP) (39). Statistics for each VOI, including volume and mean intensity values of Ktrans and rBF were calculated using the ITK-SNAP software.

MRI studies for each patient excluding TWIST sequences were interpreted by a board-certified specialist in diagnostic imaging and a primary imaging diagnosis of meningioma was given in all cases.

### Surgery

Anaesthetic protocols were assessed and tailored for each patient by the corresponding board-certified anaesthetist. Patients underwent either transfrontal craniotomy/craniectomy or a lateral rostro-tentorial craniectomy for tumour resection.

### Tissue analysis

Tumour tissue was immersed in 10% buffered formalin and processed by routine paraffin embedding. Sections were cut at 5 μm and stained with haematoxylin and eosin for histopathologic diagnosis by a board-certified pathologist.

### DCE sequence image analysis

T1 maps and DCE sequences were imported into the Nordic-ICE (NordicNeuroLab, Bergen, Norway) software package and maps of Ktrans and rBF were generated using the previously described, extended Tofts’ model (37, 40, 41). Calculation of quantitative kinetic parameters for Ktrans and rBF from DCE MRI were derived from gadolinium concentration vs. time curves (see Figure 1) fitted to the previously described two-compartment pharmacokinetic model proposed by Tofts et al. (37).

Image processing consisted of noise correction, motion artefact correction and baseline T1 correction using acquired T1 maps. The signal was normalised by an arterial input function (AIF) obtained from the selection of 10–15 voxels in the rostral cerebral artery in each patient with good signal-to-noise ratio in the canine dataset. Mean voxel-wise Ktrans and rBF values within the CE tumour VOI were calculated then averaged across all voxels with non-zero signal intensity. Zero-signal intensity voxels were removed to avoid including voxels where the model had failed to fit. This is a normal occurrence that can be caused by T2* effects and image noise that can cause an underestimation of the magnitude of the peak of the AIF in some voxels, particularly when working with high resolution images or high magnetic fields (42).

### Statistical analysis

For statistical analysis animals were divided into two groups—animals with a histopathological diagnosis of meningioma were grouped under “meningioma” and animals with a histopathological diagnosis of an intracranial neoplasm of non-meningeal origin were grouped as “other.” Each animal served as its own control, with a VOI of similar size to the CE tumour volume selected within the white matter of the contralateral side of the brain to be used as a control for normal Ktrans and rBF. This group of VOIs was termed the “control” group.

Pearson’s correlation tests were performed between CE tumour VOI and mean Ktrans and rBF values calculated within the CE tumour VOI on patients with a histopathological diagnosis of meningioma to assess the existence of linear correlations between each pair of variables, respectively. Wilcoxon matched-paired signed ranks test was performed within the meningioma group to evaluate differences in mean values of Ktrans and mean values of rBF between the CE tumour VOI and the control VOI. Finally, the Mann–Whitney U test was performed to assess differences in Ktrans and rBF values between meningioma group and the other group. All tests were one-tailed and a *p*-value of 0.05 or less was considered statistically significant. All statistical analyses were performed using GraphPad Prism 7 Software.

## Results

### Signalment

DCE-MRI and surgery with histological diagnosis was performed in ten dogs with a total of ten intracranial lesions. Patient signalment, age at presentation, tumour localization, tumour size and histopathology are summarised in Table 1. Seven dogs had a histopathological diagnosis of meningioma (Meningothelial: 1, transitional: 3, psammomatous meningioma: 1, mixed fibroblastic/epitheloid: 1, epitheloid: 1) The three dogs that had tumours which were not diagnosed as meningioma on histopathology included granular cell tumour (1), glial cell tumour (1), and histiocytic sarcoma (1).

**Table 1:** Patient signalment and histopathological diagnosis.

Animal	Age (years)	Breed	Sex	Tumour location	Histopathological diagnosis
1	9	Golden Retriever	MN	Frontal lobe	Meningothelial meningioma (atypical)
2	7	Labradoodle	FS	Frontal lobe	Histiocytic Sarcoma
3	11	Wheaten Terrier	MN	Frontal/olfactory lobe	Epitheloid Meningioma
4	11	Shetland Sheepdog	MN	Frontal lobe	Meningioma – transitional (mixed meningiothelial and fibroblastic type)
5	8	Cavoodle	MN	Temporoparietal lobe	Meningioma (mixed fibroblastic/epitheloid)
6	10	Cavoodle	MN	Frontoparietal lobe	Transitional Meningioma *
7	11	English Staffordshire Bull terrier	MN	Olfactory Lobe	Glial cell neoplasm (astrocytoma)
8	12	Labrador X	FS	Frontoparietal lobe	Psammomatous meningioma
9	7.5	Pug X	FS	Frontal lobe	Transitional meningioma
10	7	Toy poodle	FS	Rostral telencephalon	Granular cell tumour

FS, female spayed; MN, Male neutered; * denotes animals not included in final statistical analysis.

### Image analysis

One dog was excluded from the meningioma group due to difficulty obtaining arterial input function values from DCE-MRI sequences for analysis. For final analysis, the study included six dogs with meningiomas and three dogs with other intracranial neoplasms.

**Figure 6:**
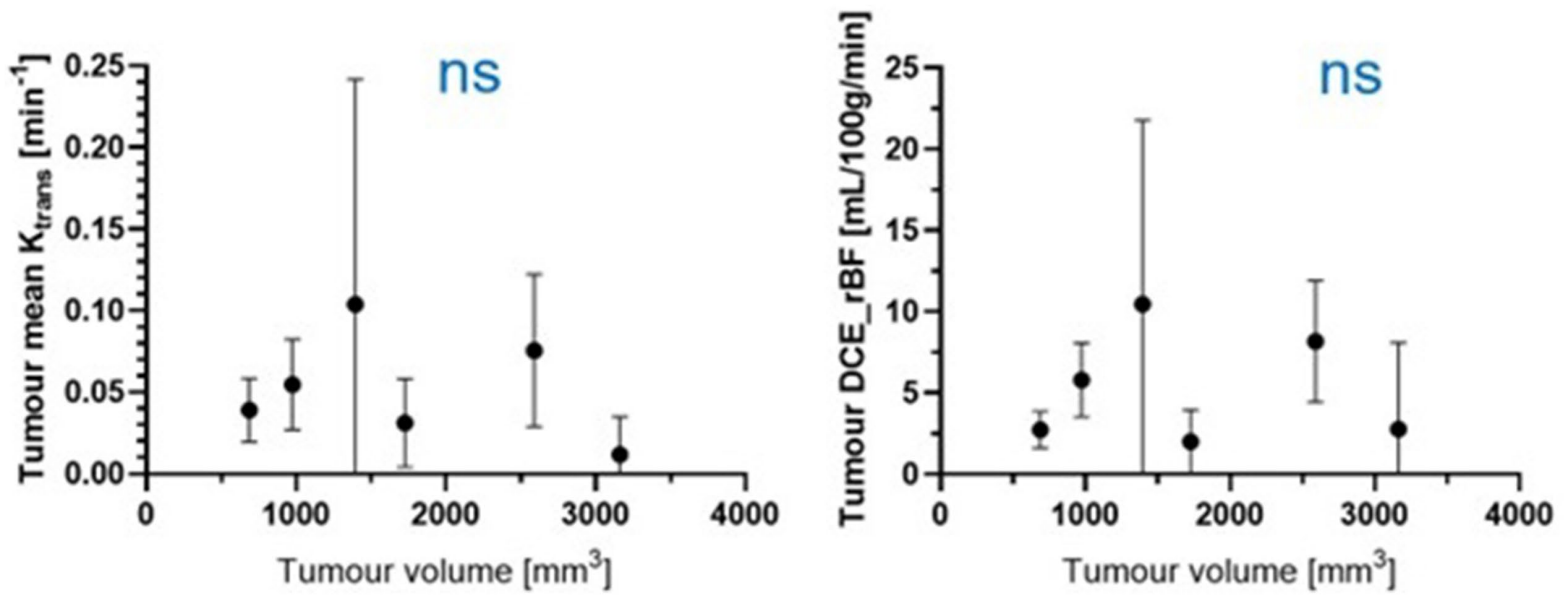
Pearson correlation. ns, not significant; *p* > 0.05.

Figure 6 shows the Ktrans and rBF of all meningioma patients. There was no statistically significant correlation between either Ktrans or rBF and tumour volume in the meningioma group using Pearson’s correlations (*r* value −0.27, *p* value: 0.5983 for Ktrans; r value −0.03565, p value: 0.9465 for rBF).

**Figure 7:**
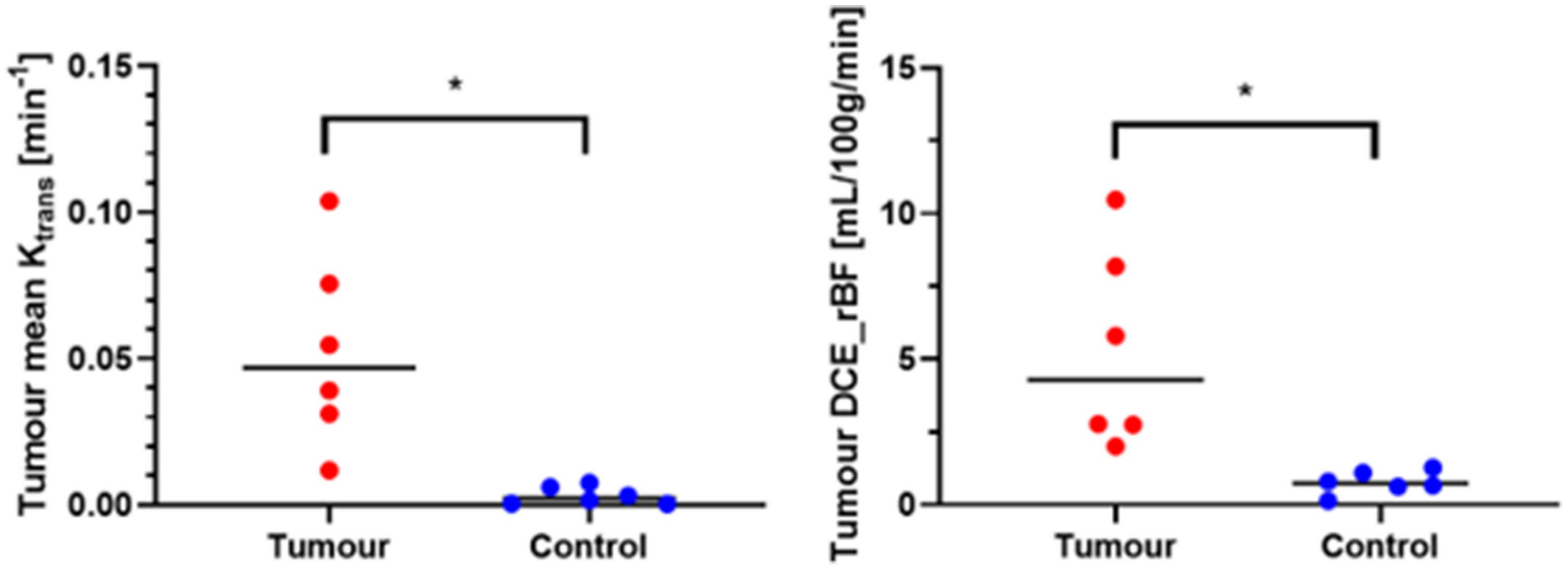
Wilcoxon matched-paired signed ranks test. *Statistically significant, *p* < 0.05; tumour, contrast enhancing tumour VOI; Control, contralateral normal brain parenchyma VOI.

Analysis of the median values of Ktrans and rBF in the CE tumour VOI and control VOI in meningioma patients are summarised in Table 2. Wilcoxon matched-paired signed ranks test revealed a statistically significant difference between the median Ktrans within CE tumour VOI compared to control VOI [−0.045 (*p* value: 0.0313)]. rBF comparisons between CE tumour VOI of meningioma compared to control VOI was also statistically different with a median of −3.824 (*p* value: 0.0313) (Figure 7). Our measurements showed that canine meningiomas demonstrated significantly higher ktrans values and rBF in contrast enhancing tumour regions when compared to contralateral parts of the brain.

**Table 2:** Ktrans and cerebral blood flow (rBF) values of CE tumour VOI vs. control VOI.

Ktrans		Cerebral blood flow (rBF)	
Tumour	Control	Tumour	Control
0.07554	0.001555	8.182527	0.661594
0.10379	0.007604	10.46861	1.27435
0.011755	0.000313	2.773495	0.127114
0.031141	0.006121	2.008031	0.623004
0.054707	0.003139	5.798886	0.797508
0.039027	0.000417	2.747369	1.087866

**Figure 8:**
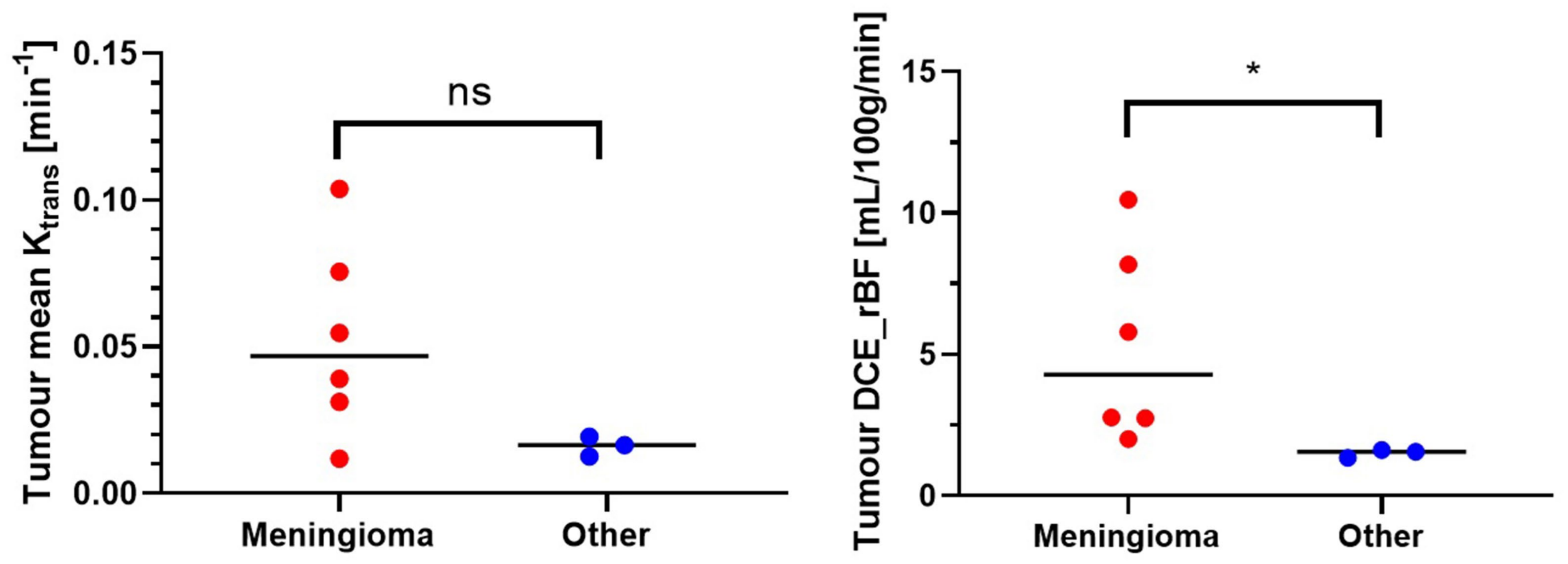
Mann–Whitney U test. ns, not significant; *p* > 0.05; *statistically significant, *p* < 0.05.

Figure 8 shows the comparison of Ktrans and rBF values between the meningioma group and other tumours. Mann–Whitney U Test revealed statistically higher rBF in the meningioma group compared to the other group (*p*-value 0.0238). Ktrans values within meningiomas were higher than other tumours however did not reach statistical significance (Table 3).

**Table 3:** Ktrans and cerebral blood flow (rBF) values of meningioma vs. other.

Ktrans		Cerebral blood flow (rBF)	
Meningioma	Other	Meningioma	Other
0.075540319	0.019257911	8.182527	1.62082
0.103790104	0.012620783	10.46861	1.347984
0.011755032	0.016456641	2.773495	1.561967
0.031140747		2.008031	
0.054707296		5.798886	
0.039027199		2.747369	

## Discussion

Advanced imaging techniques, particularly MRI has revolutionised the clinical diagnosis and management of brain tumours. In veterinary medicine MRI is routinely used to identify and classify canine brain tumours, being the preferred modality to assess for intracranial disease (4, 20, 35, 43–48). DCE-MRI is a valuable technique that characterises the movement of contrast agents from the vascular space into the extracellular space of brain tumours, thereby providing a potential method for the discrimination of tumour subtypes. The results of this study demonstrate that quantitative analysis using DCE-MRI can be used in canine patients to help differentiate meningiomas from other tumours with a similar appearance, since rBF within meningiomas was significantly higher than in the other tumour group. Ktrans values in meningiomas in this study were generally higher than other intracranial tumours, but this finding did not reach statistical significance. Finally, this study showed that meningiomas demonstrate significantly higher Ktrans and rBF values compared to normal brain parenchyma and that Ktrans values correlate poorly with tumour volume. This suggests that vascular permeability within meningiomas is not affected by the size of the tumour.

Meningiomas are extra-axial meningeal-based lesions arising from the arachnoid cap cells that line the middle layer of meninges, thus they are located in an intradural but extra-parenchymal location (7). Histologically meningiomas are characterised by pronounced interdigitations and desmosomal interconnections between neighbouring cells which are thought to reduce the likelihood of exfoliation of tumour cells. This has been suggested as the reason for the low prevalence of metastasis (49). Meningiomas, being extra-axial in nature, are not protected by the blood–brain barrier and homogenous contrast-enhancement is a consistent imaging feature observed on conventional MRI (6, 12, 13, 44, 50, 51). Although as a group meningiomas are described as highly vascular neoplasms (52), the degree of angiogenesis is likely to differ between different types of meningiomas with benign variants demonstrating less aggressive and random neovascularisation compared to more anaplastic variants. The meningiomas in this study were all histopathologically classified as benign subtypes, which could suggest that neovascularisation is rate-limited by tumour volume and that slow growth allows vessels to mature in a structured pattern. This would explain the poor correlation between tumour volume and both Ktrans and rBF values.

Blood supply to benign meningiomas is often derived from the external carotid artery via dural branches. As meningiomas enlarge, they may recruit pial branches from the brain parenchyma along the periphery of the tumour. This process of neovascularisation with the formation of immature and tortuous tumour vessels that form a dense and fragile capillary network is postulated to result in increased leakage of contrast agent from vessels into the extravascular space (53, 54). Given that meningiomas are highly vascularised and perfused extra-axial tumours with no endothelial tight junctions to serve as permeability barriers, this would account for the significantly higher values of Ktrans and rBF seen within meningiomas compared to normal brain parenchyma (52, 55). Angiogenesis, the process leading to the formation of new vessels from an existing vascular network, is essential for tumour growth (29, 52, 56). Vascular endothelial growth factor (VEGF) is an important determinant of angiogenesis. It is a potent enhancer of microvascular endothelial permeability by inducing the formation of endothelial fenestrations and opening endothelial intercellular junctions and has been found to be upregulated in canine meningiomas (52, 56–58). Differences in intra-tumoral VEGF expression have been found between different intracranial tumours in dogs with higher levels detected in astrocytic gliomas, intermediate levels in oligodendrogliomas and lowest in meningiomas (56). Apart from one dog with a histopathological diagnosis of a glial-based tumour, it is possible the remaining non-meningeal tumours in the present study exhibited lower intra-tumoural VEGF compared to meningiomas, reflecting a lower degree of tumour neovascularisation, resulting in reduced permeability and cerebral blood flow values as detected by DCE-MRI. Intra-tumoural VEGF was not measured in our patients to confirm whether kinetic parameters obtained using DCE-MRI correspond to VEGF expression, as it has been found in one human paper (59), but this would be an interesting avenue for further study.

In humans, the MRI-based predictive accuracy of diagnosing meningiomas is reported to be between 65 and 96% (60, 61), whilst in dogs the reported sensitivity of MRI to correctly identify intracranial meningiomas range from 60 to 100% (6, 13, 44). A recent study assessing interobserver reliability of experienced radiologists to correctly identify intracranial meningiomas from other extra-axial, meningeal-based tumours ranged from 79 to 94% (51).

Three dogs in this study had a histopathological diagnosis of an intracranial tumour that was not consistent with the initial imaging diagnosis of a meningioma. These included Histiocytic Sarcoma, Granular Cell Tumour and a Glial Cell Tumour. Intracranial meningiomas and histiocytic sarcomas share several overlapping MRI features. Both are intradural extra-axial neoplasms commonly presenting as a solitary meningeal-based lesion that is hyperintense on T2-weighted imaging, hypo-to iso-intense on T1-weighted imaging, contrast enhancing after administration of gadolinium-based contrast agents in addition to demonstrating a “dural tail sign” (4, 6, 12). Granular cell tumours are described as plaque-like meningeal growths that are predominantly T1w hyperintense and demonstrate strong contrast enhancement (17, 18). Along with an extra-axial anatomic location, plaque-like distribution patterns along with T1w hyperintensity have been documented for canine intracranial meningiomas (12). The third dog in the other group was histopathologically diagnosed with a glial cell neoplasm. Gliomas, in the majority of cases, can be readily differentiated from meningiomas as they originate within the brain parenchyma (i.e., intra-axial) and frequently demonstrate ring enhancement (20) as opposed to a uniform enhancement pattern that is predominantly seen in meningiomas (12). Although infrequent, uniform contrast enhancement along with an extra-axial appearance for gliomas has been described (48). In this dog a large peripherally contrast enhancing mass with an eccentrically located cystic structure within the right olfactory lobe and frontal lobe was appreciated on MRI and a primary imaging diagnosis of a cystic meningioma was given by the radiologist.

To the authors knowledge, this is the first veterinary paper analysing DCE-MRI for canine intracranial tumours using absolute quantitative analysis. Dynamic susceptibility contrast MRI (DSC-MRI) has traditionally been the method of choice for measurement of cerebral blood flow and cerebral blood volume with MRI in humans (62). There are, however, ongoing challenges associated with absolute quantification with a major source of error in DSC-MRI revolving around differences in blood and tissue relaxivity. The biggest issue arises when the blood brain barrier is damaged. Extravasation of contrast agent reduces susceptibility contrast and amplifies the T1-effects causing signal increase resulting in incorrect measurements of CBF and CBV. Several methods have been proposed to minimise this T1 interference including data truncation, correction by modelling and dual-echo sequence, however none of them reliably correct for the loss in susceptibility contrast and eliminate the error in the measurement of blood–brain barrier (BBB) leakage itself (63). DCE MRI on the other hand is more suitable when absolute quantification is required and is the method of choice for permeability measurements. The benefits of DCE-MRI also include higher spatial resolution, increased reliability of quantification measurements for microvasculature and permeability indices along with reduced susceptibility artefacts when compared to DSC-MRI (62, 64).

Although DCE-MRI has been used for several decades in humans (65–67), it is rarely utilised in veterinary medicine. Two previous studies have investigated DCE-MRI in veterinary neuro-oncology. Zhao et al. performed DCE-MRI in 7 dogs with various histologically confirmed intracranial tumours and found kinetic parameter differences in the contrast distribution pattern between tumours (35). This study predominantly used semiquantitative measures via an initial area under galodinium concentration curve. Another canine study utilising DCE-MRI to assess blood brain barrier dysfunction between extra-axial and intra-axial brain tumours highlighted a difference in the extent and distribution of BBB dysfunction between gliomas and meningiomas. This study utilised subtraction enhancement analysis (SEA), assessing the differences in gadolinium intensity signals within voxels from T1W pre-and post-contrast sequences. Along with being a semi-quantitative form of analysis, a subset of dogs used in the DCE-MRI protocol had no histopathological diagnosis and were categorised as being intra-or extra-axial based on imaging appearance (36). A significant limitation of routine clinical use of DCE-MRI is due to complex imaging requirements. DCE-MRI protocols require initiation of the dynamic sequence before intravenous contrast injection followed by repeated scanning, prolonging overall anaesthesia time. These techniques are not part of the routine protocol for standard brain MRI studies and are not available on all MRI machines, which limits their use. Separate to DCE-MRI, a small number of veterinary papers have applied advanced MRI techniques to differentiate between intracranial tumour types that otherwise have overlapping standard MRI characteristics. Diffusion-weighted imaging has demonstrated differences in apparent diffusion coefficient and fractional anisotropy to differentiate between intracranial meningiomas and histiocytic sarcomas (68). Three-dimensional time-of-flight magnetic resonance angiography in one study of dogs suggested that vessel displacement was more commonly observed with canine intracranial meningiomas than with intracranial histiocytic sarcoma (5).

There were several limitations to this study. One patient had to be excluded from the statistical analysis due to difficulty obtaining AIF for quantitative analysis and Ktrans values could not be calculated. Although a group averaged, or fixed AIF could have been used, a previous study has demonstrated significant interindividual variation in the AIF and subsequent consequences when assessing pharmacokinetics (69). In order to limit this potential by assuming a common AIF for all patients, direct measurement of the AIF was performed individually. Although quantitative models that utilise the AIF require more rigorous theory and modelling, these methods provide a more accurate reflection of physiology than semi-quantitative methods (54). As a result of this exclusion, our sample population was smaller than expected. In particular, the number of non-meningeal tumours in the ‘other’ group was small, largely because of the relatively small number of tumours that present mimicking meningiomas on imaging studies. In this study the arterial input function (AIF) was determined by averaging the signal intensity time evolution in 5 voxels in a volume of interest (VOI) placed over the rostral cerebral artery. These 5 voxels were automatically selected by the software as the 5 voxels best fitting a typical AIF signal. This method provides high signal-to-noise ratio and gives a single average value per VOI to simplify statistical analysis. The drawback of this method is that it averages voxels that may have different behaviours, leading to an average enhancement curve which may not have a real physiological basis. Another method to counteract this drawback would be voxel-by-voxel analysis, however this would require significantly greater computational power and time. Furthermore, if the signal to noise ratio is not sufficient due to the voxel’s small size, the quality of the model’s fit becomes unreliable (31).

## Conclusion

Our study demonstrated that quantitative analysis using DCE-MRI, in particular cerebral blood flow, can be utilised to differentiate canine intracranial meningiomas from other similar appearing intracranial tumours. Ktrans values were higher within meningiomas than in other types of brain tumours, however this difference did not reach statistical significance. In future studies a higher number of histopathologically confirmed meningioma cases and intracranial tumours that mimic meningiomas on MRI should be analysed with DCE-MRI to assess whether Ktrans can be used alongside rBF as an imaging biomarker for diagnosing meningiomas in dogs.

## Data Availability

The original contributions presented in the study are included in the article/supplementary material, further inquiries can be directed to the corresponding author.
